# Organic Food Consumption during Pregnancy and Hypospadias and Cryptorchidism at Birth: The Norwegian Mother and Child Cohort Study (MoBa)

**DOI:** 10.1289/ehp.1409518

**Published:** 2015-07-09

**Authors:** Anne Lise Brantsæter, Hanne Torjusen, Helle Margrete Meltzer, Eleni Papadopoulou, Jane A. Hoppin, Jan Alexander, Geir Lieblein, Gun Roos, Jon Magne Holten, Jackie Swartz, Margaretha Haugen

**Affiliations:** 1 Division of Environmental Medicine, Department of Exposure and Risk Assessment, Norwegian Institute of Public Health, Oslo, Norway; 2 National Institute for Consumer Research (SIFO), Oslo, Norway; 3 Center for Human Health and the Environment, Department of Biological Sciences, North Carolina State University, Raleigh, North Carolina, USA; 4 Office of the Director-General, Norwegian Institute of Public Health, Oslo, Norway; 5 Department of Plant Sciences, Norwegian University of Life Sciences, Ås, Norway; 6 Oikos–Organic Norway, Oslo, Norway; 7 Department of Neuroscience, Child, and Adolescent Psychiatry, Uppsala University, Uppsala, Sweden; 8 Vidarkliniken, Järna, Sweden

## Abstract

**Background:**

The etiologies of the male urogenital anomalies hypospadias and cryptorchidism remain unclear. It has been suggested that maternal diet and environmental contaminants may affect the risk of these anomalies via placental or hormonal disturbances.

**Objectives:**

We examined associations between organic food consumption during pregnancy and prevalence of hypospadias and cryptorchidism at birth.

**Methods:**

Our study includes 35,107 women participating in the Norwegian Mother and Child Cohort Study (MoBa) who delivered a singleton male infant. Information about use of six groups of organically produced food (vegetables, fruit, bread/cereal, milk/dairy products, eggs, and meat) during pregnancy was collected by a food frequency questionnaire. Women who indicated that they sometimes, often, or mostly consumed organic foods in at least one of the six food groups were classified as organic food consumers in analyses. Hypospadias and cryptorchidism diagnoses were retrieved from the Medical Birth Registry of Norway. We estimated odds ratios (ORs) and 95% confidence intervals (CIs) using multiple logistic regression.

**Results:**

Seventy-four male newborns were diagnosed with hypospadias (0.2%), and 151 with cryptorchidism (0.4%). Women who consumed any organic food during pregnancy were less likely to give birth to a boy with hypospadias (OR = 0.42; 95% CI: 0.25, 0.70, based on 21 exposed cases) than women who reported they never or seldom consumed organic food. Associations with specific organic foods were strongest for vegetable (OR = 0.36; 95% CI: 0.15, 0.85; 10 exposed cases) and milk/dairy (OR = 0.43; 95% CI: 0.17, 1.07; 7 exposed cases) consumption. No substantial association was observed for consumption of organic food and cryptorchidism.

**Conclusions:**

Consumption of organically produced foods during pregnancy was associated with a lower prevalence of hypospadias in our study population. These findings were based on small numbers of cases and require replication in other study populations.

**Citation:**

Brantsæter AL, Torjusen H, Meltzer HM, Papadopoulou E, Hoppin JA, Alexander J, Lieblein G, Roos G, Holten JM, Swartz J, Haugen M. 2016. Organic food consumption during pregnancy and hypospadias and cryptorchidism at birth: the Norwegian Mother and Child Cohort Study (MoBa). Environ Health Perspect 124:357–364; http://dx.doi.org/10.1289/ehp.1409518

## Introduction

Hypospadias and cryptorchidism are genital birth defects in male neonates. Hypospadias is a condition defined by the penile meatus not being at the tip of the penis as a result of failure of the urethral fold to unite over and cover the urethral groove. Cryptorchidism is diagnosed when one or both testicles have not descended into the scrotum (Paulozzi 1999). The prevalence of both hypospadias and cryptorchidism in Norway is around 0.3% (Aschim et al. 2004a, 2006).

The etiology of these disorders remains largely unknown, but existing evidence suggests both genetic and environmental contributors (Aschim et al. 2004b; Thorup et al. 2014). Fetal growth restriction and preeclampsia have been consistently associated with hypospadias, a finding that may implicate placental insufficiency as an underlying cause (Thorup et al. 2014). Maternal diet composition strongly influences placental function, level of inflammation and fetal nutrient supply (Thornburg et al. 2010). Other *in utero* exposures of interest in relation to anomalies of sexual maturation are substances in the environment, for example, organochlorine pesticides and other endocrine-active chemicals. There is evidence from animal studies that environmental xenohormones interfere with male genital development (Bergman et al. 2013), but the evidence from human studies is not conclusive (Carmichael et al. 2013; Rocheleau et al. 2009; Trabert et al. 2012; Virtanen and Adamsson 2012). Heavy metals, such as cadmium, have also been associated with hypospadias (Sharma et al. 2014).

The principles of organic farming as formulated by the International Federation of Organic Agriculture Movements (IFOAM) imply no use of agrochemicals (artificial pesticides, growth regulators, veterinary medicines, and synthetic soluble fertilizers) as well as no use of genetically modified organisms (Luttikholt 2007). The definitions of organic used for labeling purposes may vary among different countries and according to the specific terms used. All food sold as organic in Norway must be certified by the nonprofit organization Debio and labeled with Debio’s Ø-label (Debio 2015). This is based on an agreement with the Norwegian Food Safety Authority and ensures that regulations for organic production are met [EU (European Union) Council Regulation 2092-91 (EU 1991)]. Debio is accredited by Norwegian Accreditation according to the quality standard ISO 65/EN 45011 and by IFOAM (2015).

Organically produced food has been shown in some cases to have higher concentrations of naturally occurring plant constituents—for example, secondary plant metabolites and lower levels of cadmium and nitrate—and lower incidence of detectable pesticide residues than conventionally produced food (Barański et al. 2014; Brandt et al. 2011; Forman and Silverstein 2012; Smith-Spangler et al. 2012). Organic dairy products have been shown to contain higher levels of beneficial fatty acids and fat-soluble antioxidants compared with conventional dairy products (Benbrook et al. 2013; Huber et al. 2011). However, findings with regard to nutritional content have not been consistent among all studies, and may be partly influenced by factors that are not a consequence of organic production practices specifically. In Norway, reports from the surveillance program for pesticide residues in plant foods have shown that although the levels of detected residues were low, pesticide residues are detected almost exclusively in conventional food samples (Mattilsynet 2013).

Few studies have investigated the potential health effects of eating organic compared to conventional foods. A Danish case–control study with mothers of boys who were operated on for hypospadias and mothers of healthy boys suggested a protective association between hypospadias in the offspring and mother choosing the organic alternatives for butter and cheese (Christensen et al. 2013). We recently reported a modest but significantly lower prevalence of preeclampsia associated with frequent consumption of organic vegetables, which was observed in addition to the reduced prevalence of preeclampsia associated with a healthy diet (Torjusen et al. 2014).

The aim of this study was to examine associations between consumption of organically produced food during pregnancy and the prevalence of hypospadias and cryptorchidism at birth.

## Material and Methods

*Study population.* The data set in this study is part of the Norwegian Mother and Child Cohort Study (MoBa), a population-based prospective pregnancy cohort study conducted by the Norwegian Institute of Public Health (Magnus et al. 2006). Participants were recruited from across Norway from 1999 through 2008, and 40.6% of the invited women participated. The cohort now includes 114,500 children, 95,200 mothers, and 75,200 fathers. Women were recruited to the study by postal invitation before the routine ultrasound examination in gestational weeks 17–19. The women were asked to provide biological samples at baseline and to answer questionnaires at regular intervals during pregnancy and after birth. The data included in this study were from two questionnaires answered in gestational weeks 15 (questionnaire 1) and 22 (questionnaire 2). Questionnaire 1 was a general questionnaire covering lifestyle, background, illness, and health-related factors. Questionnaire 2 was a food frequency questionnaire (FFQ) asking the women to report average dietary intakes since the start of pregnancy. The cohort database is linked to the Medical Birth Registry of Norway (MBRN), which was established in 1967 and contains information about pregnancy, delivery, and health of the mother and the neonate for every birth or abortion after the 12th week of gestation (Irgens 2000).

The source population eligible for inclusion in the present study (*n* = 74,774) were women participating in MoBa who had responded to questionnaire 1 and questionnaire 2 and were registered in MBRN with a singleton delivery, including stillbirths. Furthermore, participants had to have answered at least one of the six questions about consumption of organic food groups in questionnaire 2 (excluding 750 participants), and they had to have reported a daily energy intake between 4.5 and 20 MJ (megajoules), excluding 1,131 participants (Meltzer et al. 2008). A total of 72,893 (97.5%) women fulfilled these criteria, of whom 37,299 delivered a male neonate. Of these, women who were included in the cohort with more than one pregnancy were included in the present study only with their first enrollment. This resulted in a final study population of 35,107 mother–infant pairs. Of these infants, 34,986 were live born, 92 were dead before delivery, 10 died during delivery, and 19 were registered with unknown time of death. There were no cases of hypospadias or cryptorchidism among the 121 babies who were not live born. The present study is based on version 4 of the quality-assured data files released for research in January 2009 (unpublished data). All MoBa participants provided written informed consent before enrollment into the study.

*Ethics approval.* MoBa was approved by the Regional Committee for Ethics in Medical Research (S-95113 and S-97045) and the Norwegian Data Inspectorate (Magnus et al. 2006).

*Data collection.* Dietary variables. Information on organic food consumption was collected by a semiquantitative FFQ which was designed specifically for assessing diet during the first 4 months of pregnancy (Norwegian Institute of Public Health 2002). The FFQ included questions about the frequency of use of organic food specified in six food groups: *a*) milk and dairy products, *b*) bread and cereal products, *c*) eggs, *d*) vegetables, *e*) fruit, and *f*) meat. There were four response categories for organic food consumption: “never/seldom,” “sometimes,” “often,” or “mostly.” Missing responses on all six group-specific questions resulted in exclusion from the study population, and missing responses on one to five questions were coded as “never/seldom.” In the present study we examined the specific organic food groups in two categories, (i.e., “never/seldom” vs. “sometimes,” “often,” and “mostly”), except for a descriptive table with three categories (“never/seldom,” “sometimes,” and “often/mostly”). The organic food groups were examined both as separate variables and as a combined variable denoting consumption of “any organic” food. The “any organic” variable was a dichotomous variable (no/yes) with yes defined as having answered “sometimes,” “often,” or “mostly” on at least one of the six organic food groups. The FFQ has been extensively validated in 119 women using a 4-day weighed food record and biological markers as reference methods. The results showed acceptable agreement (< 10% grossly misclassified and correlation coefficients ranging from 0.3 to 0.6) between the FFQ estimates and the reference methods with regard to nutrients, dietary supplements, and food groups including fruit and vegetables (Brantsaeter et al. 2007a, 2007b, 2008). The validity of the questions about organic food consumption has not been evaluated. We did not have information about quantity (grams/day) for organic foods, only frequency. From the FFQ we included the daily intakes (grams/day) of vegetables, fruit, cereals, milk/dairy, eggs, and meat, and variables denoting a vegetarian diet (vegans, lacto-vegetarians, or lacto-ovo-vegetarians, no/yes), alcohol consumption (no/yes), dietary supplement use (no/yes). To take the total diet into account we previously used principal component analysis to extract dietary patterns based on the quantitative intakes of 58 major food groups. The first principal component was interpreted as a healthy dietary pattern, with high positive loadings for vegetables, fruit and berries, plant oils, and whole grain cereals (Torjusen et al. 2012); those in the highest tertile were deemed “high scores.”

Outcome variables. Information about hypospadias and cryptorchidism was retrieved from MBRN. MBRN includes all live births and stillbirths with a gestational age > 16 weeks. The main objective of the registry is the surveillance and detection of changes in perinatal health. Notification is compulsory and is carried out by midwives or physicians attending the birth within 7 days after delivery. The standardized form contains detailed information about the parents and the child—for example, maternal health before and during index pregnancy, procedures and complications during delivery, and condition of the child at birth. Medical coding is classified according to the *International Classification of Diseases, 10th Revision* (ICD-10 codes). Hypospadias was classified with ICD-10 codes Q54.0, Q54.1, Q54.2, Q54.3, Q54.4, Q54.8, or Q54.9, and cryptorchidism was classified with ICD-10 codes Q53.0, Q53.1, Q53.2, or Q53.9. MBRN is a national birth registry that since its establishment in 1967 has been an important source for epidemiological surveillance and research (Irgens 2000). A comparison of prevalence rates of hypospadias diagnoses in MBRN and the number of surgical procedures for hypospadias performed during a year and registered in the Norwegian Patient Registry showed approximately 25% underreporting in MBRN. The authors (Aschim et al. 2004a) concluded that mild hypospadias cases are likely to be underreported in MBRN. No information is available on the performance of the registry with regard to cryptorchidism.

Other variables. In the baseline questionnaire women provided information about sociodemographic and lifestyle variables including maternal prepregnancy weight and height for calculation of body mass index (BMI), parity, level of education, household income, leisure exercise activity, and maternal as well as paternal smoking habits, use of oral contraceptives, and handling of “disinfectants or vermin poisons” or “weed killers, insecticides, or fungicides” at work or at home during the preceding 6 months. BMI was categorized according to the World Health Organization classification as normal (18.5–24.9 kg/m^2^), underweight (< 18.5 kg/m^2^), overweight (25.0–29.9 kg/m^2^), and obese (≥ 30.0 kg/m^2^). In addition, we included a missing category comprising those who had missing information on weight or height (*n* = 924). Parity was divided into two categories (nulliparous and parous). Education was divided into the following categories: high school or less (≤ 12 years), 3–4 years of college/university (13–16 years), ≥ 4 years of college/university education (≥ 17 years), and other/missing information (*n* = 924). Household income was measured as a combination of the participant’s and her partner’s income [both < 300,000 NOK (Norwegian kroner; 1 NOK ~ 0.12 GBP), one ≥ 300,000 NOK, or both ≥ 300,000 NOK]. In addition, we included a missing category (*n* = 1,025). Maternal and paternal smoking habits during pregnancy were divided into two categories, nonsmokers versus smokers.

Information about singleton or plural delivery, infant sex, maternal age at delivery, paternal age, infant birth weight, gestational age, *in vitro* fertilization (IVF), and previous stillbirths was retrieved from the MBRN. Gestational age was calculated from expected date of delivery on the basis of first-trimester ultrasound. In the event of missing an ultrasound measure, gestational age was calculated from last menstrual period. Preterm delivery was defined as gestational age < 37 weeks. Small for gestational age (SGA) was defined as infant birth weight lower than the 10th percentile for nulliparous and multiparous births for each week of gestation, based on the distribution in all singleton pregnancies in MoBa. Missing information on birth weight or gestational age and observations with birth weight < 600 g and gestational age < 25 weeks resulted in 204 infants with missing data on SGA. These were excluded in the adjusted analyses for hypospadias. Likewise, 123 missing data on paternal age were excluded in the adjusted analyses for cryptorchidism.

*Statistical analyses.* All *p-*values were two-sided, and values < 0.05 were considered statistically significant. The Pearson chi-square test was used to test for group difference in categorical data.

Crude and adjusted odd ratios (OR) with 95% confidence intervals (CIs) were estimated for the association between consumption of *a*) any organic food expressed in a combined variable of all organic food groups and the outcomes, *b*) individual organic food groups and the outcomes, and *c*) individual food groups and the outcomes with additional adjustment for use of any organic food.

Characteristics and dietary quality associated with organic food consumption in MoBa have been described in detail previously (Torjusen et al. 2010, 2012). Variables considered as potential confounding variables in the current study were maternal age, paternal age, maternal prepregnancy BMI, parity, maternal education, leisure exercise activity, being a student, household income, dietary supplement use, having a vegetarian diet, healthy diet scores, energy intake, use of alcohol, maternal smoking, paternal smoking, handling of “disinfectants or vermin poisons” or “weed killers, insecticides, or fungicides” at work or at home during the previous 6 months, IVF, oral contraceptives any time during the previous year, previous stillbirths, preterm delivery, and SGA. From these, variables were selected on the basis of their association with both the exposure and the outcome (*p* < 0.10), using any organic variable as the exposure. Variables fulfilling this criterion with hypospadias as outcome were prepregnancy BMI, maternal education, leisure exercise activity, and household income. Variables fulfilling this criterion with cryptorchidism as outcome were paternal age, maternal education, mother being a student, and household income. In addition, we included known risk factors for hypospadias or cryptorchidism (IVF, preterm delivery, and SGA). Adjustment variables were assessed using the change in estimate method, starting with all variables in the models with deletion of one by one in a stepwise manner (backward regression). None of the variables resulted in a change in estimate > 10%, and all variables with *p* < 0.10 were retained in the final models. The variables retained in the analyses of hypospadias were maternal education, household income, prepregnancy BMI, SGA, and preterm delivery. The variables retained in the analyses of cryptorchidism were maternal education, household income, and paternal age. In addition, the calculated amount of vegetables, fruit, cereals, milk/dairy, eggs, and meat was included in the model for each organic food group to adjust for quantity although these did not meet the criterion of *p* < 0.10.

All analyses were performed using the statistical package for social sciences version 20.0 (IBM SPSS Statistics 20) for Windows (SPSS, Chicago, IL, USA).

## Results

Of the 35,107 mothers in this study, 17,996 (51.3%) reported “never/seldom” use of all the organic food, 11,370 (32.4%) reported “sometimes” as the highest frequency for at least one organic group, and 5,741 (16.4%) reported “often or mostly” for at least one of the six organic food groups. The number of neonates with hypospadias was 74 (0.2%) and the number with cryptorchidism was 151 (0.4%) diagnosed within 7 days of birth. Only one boy had both anomalies. Use of any organic food was associated with higher education and household income, and with lower BMI, nulliparity, nonsmoking, being a student, use of dietary supplements, drinking alcohol, adhering to vegetarian diet, and having a healthy diet (Table 1). In general the percentages reporting “never/seldom” consumption of organic food were higher in hypospadias cases than in the total study population, whereas this was not seen for cryptorchidism (Table 2). The most widely consumed organic food group was vegetables, with 35.2% of women reporting this “sometimes,” “often,” or “mostly,” followed by eggs (34.1%), fruit (28.8%), milk/dairy products (26.0%), cereals (20.3%), and meat (12.2%). The highest proportion of organic consumers was found for the vegetable group (72.2%), but there were substantial overlap among the organic food groups (Table 3).

**Table 1:** Organic food consumption,*^a^* by maternal characteristics [*n* (%)].

Characteristic	All [35,107 (100)]	Never/seldom organic [17,996 (51.3)]	Sometimes, often, or mostly organic [17,111 (48.7)]	*p*‑Value^*b*^
Maternal age (years)				< 0.001
< 20	667 (1.9)	310 (46.5)	357 (53.5)
20–29	18,041 (51.4)	9,624 (53.3)	8,417 (46.7)
≥ 30	16,399 (46.7)	8,062 (49.2)	8,337 (50.8)
Paternal age (years)				< 0.001
< 20	184 (0.5)	79 (42.9)	105 (57.1)
20–29	12,223 (34.8)	6,492 (53.1)	5,731 (46.9)
≥ 30	22,577 (64.3)	11,375 (50.4)	11,202 (49.6)
Missing information	123 (0.4)	50 (40.7)	73 (59.3)
Maternal BMI (kg/m^2^)				< 0.001
< 18.5	1,017 (2.9)	516 (50.7)	501 (49.3)
18.5–24.9	22,336 (63.6)	11,123 (49.8)	11,213 (50.2)
25–29.9	7,500 (21.4)	4,055 (54.1)	3,445 (45.9)
≥ 30	3,330 (9.5)	1,826 (54.8)	1,504 (45.2)
Missing information	924 (2.6)	476 (51.5)	448 (48.5)
Parity				< 0.001
Nulliparous	16,919 (48.2)	8,502 (50.3)	8,417 (49.7)
Parous	17,669 (50.3)	9,211 (52.1)	8,458 (47.9)
Missing information	519 (1.5)	283 (54.5)	236 (45.5)
Maternal education (years)				< 0.001
≤ 12	11,099 (31.6)	6,235 (56.2)	4,464 (43.8)
13–16	17,793 (42.1)	7,741 (52.3)	7,052 (47.7)
≥ 17	8,448 (24.1)	3,605 (42.7)	4,843 (57.3)
Missing information	767 (2.2)	415 (54.1)	352 (45.9)
Mother student				< 0.001
No	31,707 (90.3)	16,433 (51.8)	15,274 (48.2)
Yes	3,400 (9.7)	1,563 (46.0)	1,837 (54.0)
Household income (NOK)				< 0.001
Both partners < 300,000	10,143 (28.9)	5,399 (53.2)	4,744 (46.8)
One partner ≥ 300,000	14,832 (42.2)	7,788 (52.5)	7,044 (47.5)
Both partners ≥ 300,000	9,107 (25.9)	4,311 (47.3)	4,796 (52.7)
Missing information	1,025 (2.9)	498 (48.6)	527 (51.4)
Dietary supplement use				< 0.001
No	7,170 (20.4)	4,123 (57.5)	3,047 (42.5)
Yes	27,937 (79.6)	13,873 (49.7)	14,064 (50.3)
Vegetarian diet				< 0.001
No	35,044 (99.8)	17,982 (51.3)	17,062 (48.7)
Yes	63 (0.2)	14 (22.2)	49 (77.8)
Healthy diet scores				< 0.001
Lowest tertiles	23,405 (66.7)	13,713 (58.6)	9,692 (41.4)
Upper tertile	11,702 (33.3)	4,283 (36.6)	7,419 (63.4)
Alcohol in pregnancy				< 0.001
No	31,106 (88.6)	16,146 (51.9)	14,960 (48.1)
Yes	4,001 (11.4)	1,850 (46.2)	2,151 (53.8)
Smoking in pregnancy				< 0.001
No	32,194 (91.7)	16,332 (50.7)	15,862 (49.3)
Yes	2,913 (8.3)	1,664 (57.1)	1,249 (42.9)
^***a***^Consumption of organic food was defined as having answered “sometimes,” “often,” or “mostly” for at least one of the six organic food groups (vegetables, fruits, cereals, milk/dairy, eggs, meat). Nonconsumption was defined as having answered “never/seldom” for all organic food groups. No data were missing on use of organic food. ^***b***^*p*‑Value obtained by Pearson’s chi-square test.				

**Table 2:** Prevalence of hypospadias and cryptorchidism diagnosed within 7 days of birth, by organic food consumption [*n* (%)].*^a^*

Organic food consumption	Total (*n* = 35,107)	Hypospadias (*n* = 74)	Cryptorchidism (*n* = 151)
Organic vegetables
Never/seldom	22,759 (64.8)	63 (85.1)	98 (64.9)
Sometimes	9,785 (27.9)	8 (10.8)	44 (29.1)
Often or mostly	2,563 (7.3)	3 (4.1)	9 (6.0)
Organic fruit
Never/seldom	25,006 (71.2)	61 (82.4)	107 (70.9)
Sometimes	7,802 (22.2)	9 (12.2)	36 (23.8)
Often or mostly	2,299 (6.6)	4 (5.4)	8 (5.3)
Organic cereals
Never/seldom	27,980 (79.7)	64 (86.5)	123 (81.5)
Sometimes	4,915 (14.0)	6 (8.1)	18 (11.9)
Often or mostly	2,212 (6.3)	4 (5.4)	10 (6.6)
Organic milk/dairy products
Never/seldom	25,992 (74.0)	67 (90.5)	120 (79.5)
Sometimes	6,582 (18.8)	5 (6.8)	18 (11.9)
Often or mostly	2,533 (7.2)	2 (2.7)	13 (8.6)
Organic eggs
Never/seldom	23,144 (65.9)	58 (78.4)	99 (65.6)
Sometimes	8,749 (24.9)	8 (10.8)	38 (25.2)
Often or mostly	3,214 (9.2)	8 (10.8)	14 (9.3)
Organic meat
Never/seldom	30,814 (87.8)	70 (94.6)	136 (90.1)
Sometimes	2,793 (8.0)	2 (2.7)	13 (8.6)
Often or mostly	1,500 (4.2)	2 (2.7)	2 (1.3)
^***a***^No data were missing on use of organic food.			

**Table 3:** Pattern of organic food consumption within the six organic food groups as reported by 35,107 pregnant women in the Norwegian Mother and Child Cohort Study (MoBa) who delivered a singleton male infant in years 2002 to 2008.

Organic food group	*n* (%)	Any organic^*a*^	
		Never/seldom	Sometimes, often, or mostly
Organic vegetables
Never/seldom	22,759 (64.8)	17,996	4,763 (27.8)
Sometimes, often, or mostly	12,348 (35.2)	0	12,348 (72.2)
Organic fruit
Never/seldom	25,006 (71.2)	17,996	7,010 (41.0)
Sometimes, often, or mostly	10,101 (28.8)	0	10,101 (59.0)
Organic cereals
Never/seldom	27,980 (79.7)	17,996	9,015 (58.3)
Sometimes, often, or mostly	7,127 (20.3)	0	7,127 (41.7)
Organic milk/dairy products
Never/seldom	25,992 (74.0)	17,996	7,996 (46.7)
Sometimes, often, or mostly	9,115 (26.0)	0	9,115 (53.3)
Organic eggs
Never/seldom	23,144 (65.9)	17,996	5,148 (30.1)
Sometimes, often, or mostly	11,963 (34.1)	0	11,963 (69.9)
Organic meat
Never/seldom	30,814 (87.8)	17,996	12,818 (74.9)
Sometimes, often, or mostly	4,293 (12.2)	0	4,293 (25.1)
^***a***^Any organic was defined as having answered “sometimes,” “often,” or “mostly” for any of the six organic food groups (vegetables, fruits, cereals, milk/dairy, eggs, meat). Nonconsumption was defined as having answered “never/seldom” for all organic food groups. No data were missing on use of organic food.			

In unadjusted models, a lower prevalence of hypospadias was seen for women who reported use of organic vegetables and organic milk/dairy products (Table 4). Adjusted ORs were similar to crude ORs (Table 4). The adjusted OR for hypospadias in association with consumption of any organic food was 0.42 (95% CI: 0.25, 0.70), based on 21 exposed cases, and significant negative associations were also estimated for consumption of organic vegetables (10 exposed cases), organic fruit (13 exposed cases), organic milk/dairy products (7 exposed cases), and organic eggs (16 exposed cases) (Table 4). Excluding observations with missing information on SGA (*n* = 204) led to exclusion of two boys with hypospadias. A total of 2,582 (7.3%) women had missing values on maternal prepregnancy BMI, educational attainment, and/or household income. When these were excluded rather than modeled as “missing” categories, the number of hypospadias cases was 68, but the associations did not change (OR = 0.42; 95% CI: 0.25, 0.71, based on 20 exposed cases). Additional adjustment for the estimated total daily intake (organic and nonorganic) of food items (grams/day) within each organic food group had no influence on the ORs.

**Table 4:** Associations between organic food consumption and hypospadias.*^a^*

Exposure to organic food	Total (*n *= 35,107)	Hypospadias *n* (%)	Crude OR (95% CI)	Adjusted^*b *^OR (95% CI)	Adjusted^*c *^OR (95% CI)
Any organic food^*d*^
Never/seldom	17,996	52 (0.3)	1	1	NA
Sometimes, often, or mostly	17,111	22 (0.1)	0.44 (0.27, 0.73)	0.42 (0.25, 0.70)
Organic vegetables
Never/seldom	22,759	63 (0.3)	1	1	1
Sometimes, often, or mostly	12,348	11 (0.1)	0.32 (0.17, 0.61)	0.30 (0.15, 0.58)	0.36 (0.15, 0.85)
Organic fruit
Never/seldom	25,006	61 (0.2)	1	1	1
Sometimes, often, or mostly	10,101	13 (0.1)	0.53 (0.29, 0.96)	0.54 (0.30, 0.99)	1.15 (0.48, 2.79)
Organic cereals
Never/seldom	27,980	64 (0.2)	1	1	1
Sometimes, often, or mostly	7,127	10 (0.1)	0.61 (0.32, 1.19)	0.62 (0.32, 1.22)	1.27 (0.54, 3.00)
Organic milk/dairy products
Never/seldom	25,992	67 (0.3)	1	1	1
Sometimes, often, or mostly	9,115	7 (0.1)	0.30 (0.14, 0.65)	0.30 (0.14, 0.65)	0.43 (0.17, 1.07)
Organic eggs
Never/seldom	23,144	58 (0.3)	1	1	1
Sometimes, often, or mostly	11,963	16 (0.1)	0.53 (0.31, 0.93)	0.54 (0.31, 0.94)	1.40 (0.51, 3.82)
Organic meat
Never/seldom	30,814	70 (0.2)	1	1	1
Sometimes, often, or mostly	4,293	4 (0.1)	0.41 (0.15, 1.22)	0.42 (0.15, 1.17)	0.72 (0.23, 2.15)
NA, not applicable. ^***a***^Numbers of observations (total and cases) do not account for observations with missing covariate data (204 missing including 2 cases). ^***b***^Results from logistic regression models adjusted for maternal education, household income, maternal prepregnancy BMI, SGA baby, preterm delivery. In addition, the model for each organic food group was adjusted for the total daily intake of food items (organic and nonorganic) in the respective group. ^***c***^Additional adjustment for consumption of any organic food. ^***d***^Reported use of at least one of the organic food groups. No data were missing on use of organic food.					

Because of the large overlap between consumption of organic food in the various food groups, the separate food groups could not be included in the same model. However, we added to the model for each of the food groups the variable “any organic use” to control for whether being an “organic consumer” influenced the results. As expected, this largely removed the associations between the organic food groups and hypospadias, except for the ORs for organic vegetables and organic milk. The association remained significant for consumption of organic vegetables, with adjusted OR = 0.36 (95% CI: 0.15, 0.85; *n* = 10 exposed cases). For organic milk/dairy products, the adjusted OR was borderline significant with OR = 0.43 (95% CI: 0.17, 1.07, *p* = 0.070; *n* = 7 exposed cases).

Consumption of organic food was not associated with the prevalence of cryptorchidism for any of the food groups (Table 5). However, a borderline negative association with cryptorchidism was found for organic milk/dairy products with OR = 0.65 (95% CI: 0.40, 1.04, *p* = 0.071; *n* = 31 exposed cases).

**Table 5:** Associations between organic food consumption and cryptorchidism.*^a^*

Exposure to organic food	Total (*n* = 35,107)	Cryptorchidism *n* (%)	Crude OR (95% CI)	Adjusted^*b*^ OR (95% CI)	Adjusted^*c*^ OR (95% CI)
Any organic food^*d*^
Never/seldom	17,996	79 (0.4)	1	1	NA
Sometimes, often, or mostly	17,111	72 (0.4)	0.96 (0.70, 1.32)	0.91 (0.66, 1.26)
Organic vegetables
Never/seldom	22,759	98 (0.4)	1	1	1
Sometimes, often, or mostly	12,348	53 (0.4)	0.99 (0.71, 1.39)	0.92 (0.66, 1.30)	1.03 (0.61, 1.74)
Organic fruit
Never/seldom	25,006	107 (0.4)	1	1	1
Sometimes, often, or mostly	10,101	44 (0.5)	1.02 (0.72, 1.45)	1.04 (0.73, 1.44)	1.17 (0.73, 1.90)
Organic cereals
Never/seldom	27,980	123 (0.4)	1	1	1
Sometimes, often, or mostly	7,127	28 (0.4)	0.89 (0.59, 1.35)	0.86 (0.57, 1.30)	0.88 (0.55, 1.42)
Organic milk/dairy products
Never/seldom	25,992	120 (0.5)	1	1	1
Sometimes, often, or mostly	9,115	31 (0.3)	0.74 (0.50, 1.09)	0.70 (0.47, 1.05)	0.65 (0.40, 1.04)
Organic eggs
Never/seldom	23,144	99 (0.4)	1	1	1
Sometimes, often, or mostly	11,963	52 (0.5)	1.02 (0.73, 1.42)	0.97 (0.69, 1.36)	1.09 (0.65, 1.82)
Organic meat
Never/seldom	30,814	136 (0.4)	1	1	1
Sometimes, often, or mostly	4,293	15 (0.4)	0.79 (0.46, 1.35)	0.78 (0.45, 1.33)	0.78 (0.44, 1.38)
NA, not applicable. ^***a***^Numbers of observations (total and cases) do not account for observations with missing covariate data (123 missing including 1 case). ^***b***^Results from logistic regression models adjusted for maternal education, household income, and paternal age. In addition, the model for each organic food group was adjusted for the total daily intake of food items (organic and nonorganic) in the respective group. ^***c***^Additional adjustment for any organic food. ^***d***^Reported use of at least one of the organic food groups. No data were missing on use of organic food.					

Finally, we repeated the analyses for any organic food consumption and hypospadias adjusting for preeclampsia (*n* = 1,387). The adjusted OR remained unchanged (OR = 0.42; 95% CI: 0.25, 0.69; *n* = 21 exposed cases). When mothers with preeclampsia were excluded from the analysis, the adjusted OR was 0.35 (95% CI: 0.20, 0.62; *n* = 16 exposed cases).

## Discussion

The main finding of this study was that women who reported “sometimes, often, or mostly” eating organically produced food during pregnancy were less likely to give birth to a boy with hypospadias than women who reported never or seldom consuming organic foods. Of the six individual organic food groups queried, the negative association was strongest for consumption of organic vegetables, based on 10 cases classified as consumers and 62 cases classified as nonconsumers. The associations were consistent and marginally influenced by adjusting for food intakes, lifestyle factors, and sociodemographic variables. However, in most cases there were substantial changes with adjustment for “any organic consumption” although the estimates are difficult to interpret due to probable collinearity. We found no association between organic food consumption and cryptorchidism, with the exception of a borderline significant association for use of organic milk/dairy products (OR = 0.65; 95% CI: 0.40, 1.04; *p* = 0.071; *n* = 31 exposed cases).

To the best of our knowledge, this is the first prospective study to report a significant association between consumption of organic food and hypospadias. There are limited data to explain this finding. Properties of organic food, particularly vegetables, which could possibly contribute to explaining the finding of a reduced prevalence of hypospadias at birth in our study population, include compositional differences with regard to bioactive substances including nutrients and pesticide residues (Barański et al. 2014; Brandt et al. 2011; Forman and Silverstein 2012; Smith-Spangler et al. 2012). However, findings with regard to nutritional content have not been consistent among all studies. Given differences in bacterial communities on the surfaces on organic and conventional fresh fruits and vegetables (Leff and Fierer 2013), one might speculate that this could affect the gut microbiota and thereby the proneness to inflammation (Bengmark 2013), but this is largely hypothetical.

Exposure to pesticides in the general population, except for occupational and accidental exposure, is mainly via residues on food (Lu et al. 2008). Consumption of organic food has been associated with lower urinary concentrations of pesticide metabolites in children and adults (Curl et al. 2003; Lu et al. 2006; Oates et al. 2014). Pesticide exposure could hypothetically lead to hypospadias via hormonal or placental disturbances. Hypospadias and cryptorchidism are both related to androgen receptor function (Toppari et al. 2010), and the role of fetal androgens is especially important during the first trimester, when organogenesis takes place (Kalfa et al. 2009). Numerous pesticides are known to be endocrine-active substances with estrogenic or anti-androgenic activity (Bergman et al. 2013). Despite restriction for use in most developed countries, organochlorine pesticides are persistent and remain in the environment together with their breakdown products (Clarke et al. 2010; Kannan et al. 1997). However, use still remains in other parts of the world. Because the Norwegian food supply has global sources, residents may be exposed to banned pesticides through consumption of imported fruits and vegetables. Metabolites of the banned pesticides DDT, hexachlorobenzene, β-hexachlorocyclohexane, and oxychlordane have been detected and quantified in Norwegian breast milk sampled in years 2002–2006 (Polder et al. 2009).

Placental insufficiency may play an important role in hypospadias etiology as fetal growth restriction and preeclampsia both are risk factors for hypospadias (Akre et al. 1999; Thorup et al. 2014; van der Zanden et al. 2012). Placental function may link the lower prevalence of hypospadias with organic vegetable consumption in the present study with our previous finding of lower prevalence of preeclampsia associated with consumption of organic vegetables (Torjusen et al. 2014). In the present study, the significant association between any organic food and lower prevalence of hypospadias remained both when adjusting for and when excluding women with preeclampsia.

We found lower prevalence of hypospadias associated with consumption of organic food, particularly organic vegetables, but it was not possible to distinguish clearly between the organic food groups. We had no data to evaluate the quantitative amount of organic food consumption because the estimated intake within the food groups comprised both organic and nonorganic items. The weak association between consumption of organic milk/dairy products and hypospadias in our study (*p* = 0.07, *n* = 7 exposed cases) is in line with the results from a case–control study in Denmark. The study included 306 boys with hypospadias and 306 controls and showed an association between hypospadias in the offspring and the mother not choosing the organic alternative, and having a high intake (≥ daily) of nonorganic butter and cheese in comparison with mothers regularly choosing the organic alternative and having a low intake of butter and cheese (< daily) (Christensen et al. 2013). The authors explained their finding by suggesting that conventionally produced butter and cheese contain more traces of pesticide residues than organic food (Christensen et al. 2013).

The strengths of this study include the prospective design and the large study population comprising pregnant women from all regions of Norway, representing all age groups and all socioeconomic groups. Information about maternal diet and use of organically produced food covered the first half of pregnancy and was assessed using a validated FFQ (Brantsaeter et al. 2008). Although many women adopt healthier lifestyle and dietary habits during pregnancy (Meltzer et al. 2008), use of organic food is likely to reflect a more long-term practice (Codron et al. 2006). The participation rate in MoBa is 40.6%, and MoBa participants are older, have higher educational attainment, and comprise fewer smokers than the general population of pregnant women. However, evaluation of this nonrepresentativeness in MoBa showed that it did not affect exposure–outcome associations, including prenatal smoking and birth outcomes (low birth weight, placental abruption, stillbirth), chronic hypertension and gestational diabetes, maternal vitamin use and placental abruption, parity and preeclampsia, marital status and preterm birth (Nilsen et al. 2009), and perinatal and prenatal exposures [primipara pregnancy (no/yes), prenatal folic acid use (no/yes), prenatal smoking (no/yes), low birthweight (no/yes), preterm birth (no/yes), offspring sex (female/male), and cesarean section history (no/yes)], and specialist-confirmed diagnosis of autism spectrum disorders (Nilsen et al. 2013).

Although our study benefits from a large cohort design, it is somewhat limited by the lack of detail regarding specific aspects of our hypothesis. We had no biological or environmental measurements to assess whether women who consumed organically produced food had different exposure to adverse or favorable substances than those who did not have organic diets. Additionally we lacked detail on the extent of organic consumption of an individual, so women who ate exclusively organic were combined with those who ate mostly conventional diets. We had no family data on genital malformations, which are known to have a hereditary component. Furthermore, use of medical registry data is likely to result in underreporting of mild forms—that is, cryptorchidism with spontaneous descent and mild hypospadias with normal foreskin. The prevalence of hypospadias in our study is similar to the prevalence in MBRN (0.2%) in mothers not exposed to anti-epileptic drugs (Veiby et al. 2014), whereas the prevalence of cryptorchidism (0.4%) was slightly higher than previously reported in Norway (0.3%) (Aschim et al. 2006). Although case diagnoses were based on international case definitions, misclassification or misreporting may occur. Ideally, case diagnoses should be ascertained through the patient registry using both diagnoses at birth and surgical operations for the condition as criteria of being a case. For the present study we did not have access to the patient registry, but a previous study that evaluated the prevalence rates for hypospadias in MBRN and the patient registry indicated misclassification primarily of mild hypospadias (Aschim et al. 2004a). Our study included 74 mothers of boys with hypospadias, of whom 22 were consumers of any organic food; and although misclassification might be random with regard to exposure, the consequences of bias cannot be predicted with certainty when the numbers of observations are small. The diagnosis of cryptorchidism is less certain because in mild cases testes descend spontaneously after birth. It is possible that misclassification of cryptorchidism might have contributed to the finding of no associations between organic foods and this outcome.

We were able to adjust for many potential confounding variables and adjustment had little influence on the results. However, use of organic food was self-reported, and residual confounding cannot be excluded. Use of organic food may reflect other lifestyle factors such as differences in use and sources of cosmetics, household cleaning products, cleaning frequency, home interior materials, clothing material, and home cooking practices (e.g., use of plastic storage containers).

In conclusion, mothers who reported “sometimes, often, or mostly” consuming organic foods during pregnancy were less likely to give birth to a boy with hypospadias than women who reported never or seldom consuming organic foods. The association between organic food consumption and lower prevalence of hypospadias were strongest for organic vegetables and organic milk and dairy products, though findings were based on small numbers of cases. We did not find evidence of an association between organic food consumption and cryptorchidism at birth in our study population. To improve public health, pregnant women are encouraged to eat more vegetables regardless of how they are produced, and choosing the organic alternative might give additional benefits. However, the replication of our findings in other cohorts is warranted.
